# Prospective Surveillance for Cardiac Adverse Events in Healthy Adults Receiving Modified Vaccinia Ankara Vaccines: A Systematic Review

**DOI:** 10.1371/journal.pone.0054407

**Published:** 2013-01-17

**Authors:** Marnie L. Elizaga, Sandhya Vasan, Mary A. Marovich, Alicia H. Sato, Dale N. Lawrence, Bernard R. Chaitman, Sharon E. Frey, Michael C. Keefer, MVA Cardiac Safety Working Group

**Affiliations:** 1 Vaccine and Infectious Disease Division, Fred Hutchinson Cancer Research Center, Seattle, Washington, United States of America; 2 Aaron Diamond AIDS Research Center, New York, New York, United States of America; 3 United States Military HIV Research Program, Walter Reed Army Institute of Research, Rockville, Maryland, United States of America; 4 Statistical Center for HIV/AIDS Research and Prevention (SCHARP), Fred Hutchinson Cancer Research Center, Seattle, Washington, United States of America; 5 Division of AIDS, National Institute of Allergy and Infectious Diseases, National Institutes of Health, Bethesda, Maryland, United States of America; 6 Division of Cardiology, Saint Louis University School of Medicine, St. Louis, Missouri, United States of America; 7 Clinical Research Division of Infectious Diseases, Allergy and Immunology, Saint Louis University School of Medicine, St. Louis, Missouri, United States of America; 8 University of Rochester School of Medicine and Dentistry, Rochester, New York, United States of America; World Health Organization, Switzerland

## Abstract

**Background:**

Vaccinia-associated myo/pericarditis was observed during the US smallpox vaccination (DryVax) campaign initiated in 2002. A highly-attenuated vaccinia strain, modified vaccinia Ankara (MVA) has been evaluated in clinical trials as a safer alternative to DryVax and as a vector for recombinant vaccines. Due to the lack of prospectively collected cardiac safety data, the US Food and Drug Administration required cardiac screening and surveillance in all clinical trials of MVA since 2004. Here, we report cardiac safety surveillance from 6 phase I trials of MVA vaccines.

**Methods:**

Four clinical research organizations contributed cardiac safety data using common surveillance methods in trials administering MVA or recombinant MVA vaccines to healthy participants. ‘Routine cardiac investigations’ (ECGs and cardiac enzymes obtained 2 weeks after injections of MVA or MVA-HIV recombinants, or placebo-controls), and ‘Symptom-driven cardiac investigations’ are reported. The outcome measure is the number of participants who met the CDC-case definition for vaccinia-related myo/pericarditis or who experienced cardiac adverse events from an MVA vaccine.

**Results:**

Four hundred twenty-five study participants had post-vaccination safety data analyzed, 382 received at least one MVA-containing vaccine and 43 received placebo; 717 routine ECGs and 930 cardiac troponin assays were performed. Forty-five MVA recipients (12%) had additional cardiac testing performed; 22 for cardiac symptoms, 19 for ECG/laboratory changes, and 4 for cardiac symptoms with an ECG/laboratory change. No participant had evidence of symptomatic or asymptomatic myo/pericarditis meeting the CDC-case definition and judged to be related to an MVA vaccine.

**Conclusions:**

Prospective surveillance of MVA recipients for myo/pericarditis did not detect cardiac adverse reactions in 382 study participants.

**Trial Registration:**

ClinicalTrials.gov NCT00082446 NCT003766090 NCT00252148 NCT00083603 NCT00301184 NCT00428337

## Introduction

Modified vaccinia Ankara (MVA) is a highly attenuated strain of vaccinia virus derived from the replication-competent Ankara vaccinia strain, chorioallantois vaccinia Ankara (CVA) [1], and was administered as a priming smallpox vaccine to over 120,000 people in Germany in the 1970s without significant side effects [2], [3]. In contrast, replication-competent vaccinia was administered to one-third of the world’s population during the World Health Organization’s (WHO) smallpox eradication program [4] and has well-recognized side-effects [5], although cardiac complications were rare in the US compared to Europe and Australia, presumably due to differences in the vaccinia strains employed [6]. Since the global eradication of smallpox in 1980, replication-competent vaccinia continued to be used as a vector for experimental vaccines [7]–[9]. However, attenuated poxviruses (MVA [10]–[11], NYVAC, canarypox and fowlpox) have subsequently gained favor as safer vectors for vaccines against multiple pathogens [12]–[16].

Concerned about potential bioterrorism, in 2002–2004 the US Department of Defense (DoD) and Department of Health and Human Services (DHHS) initiated a smallpox vaccination campaign with the New York City Board of Health (NYCBOH) vaccinia strain (DryVax, Wyeth Laboratories Inc, Marietta, PA) to protect military personnel and civilian first-responders. By 2005, approximately 39,500 civilians and 730,500 military personnel had been vaccinated [17] and use in the military has continued with 2.2 million people vaccinated to date [18].

Shortly after the US campaign began, several cases of myo/pericarditis were reported among primary vaccines [6], [19]–[21] and revaccinees [6]–[19] and the US Centers for Disease Control (CDC) released a warning on March 28, 2003 [22]. Cases typically presented 1–2 weeks after receipt of DryVax with acute chest pain, dyspnea, or palpitations, ECG changes, elevated cardiac enzymes, depressed left ventricular function and/or abnormal cardiac imaging indicating myocardial inflammation. By the end of 2003, 67 cases of myo/pericarditis were reported among 540,824 vaccinees (12.4 cases per 100,000 vaccinees [or 1 case per 8065 vaccinees]) in the DoD program [21] and 21 cases among 37,901 vaccinees (550 cases per 100,000 vaccinees [or 1 case per 182 vaccinees]) in the DHHS program [6]. In both groups, the clinical course of myo/pericarditis was generally mild or moderate, with full recovery the most frequent outcome. Consequently, the US FDA required prospective cardiac monitoring in a phase III trial comparing replication-competent vaccinia produced in cell culture, ACAM2000 (Sanofi Pasteur, Swiftwater, PA [formerly Acambis]) to DryVax. Ten cases of myo/pericarditis occurred among 1675 vaccinia-naïve participants receiving either vaccine (1 case per 168 vaccinees), with no significant difference in incidence between the two preparations and importantly, about half of these events were subclinical, detected only by ECG or cardiac troponin abnormalities [17]–[23]. While the mechanism of cardiotoxicity is unclear, limited published data suggests an immunologically-mediated rather than a direct cytotoxic effect [24].

Although MVA’s replication in mammalian cells is limited [25] and it is avirulent in immunosuppressed animals [3], [26], it remains unclear whether it can induce myo/pericarditis. Consequently the FDA extended the requirement for prospective cardiac monitoring to clinical trials employing MVA. In order to investigate the rate of vaccine-related cardiac adverse events from investigational MVA vaccines in Phase I trials, we review the cumulative experience of 4 clinical research organizations that employed uniform methods of prospective cardiac monitoring in 6 clinical trials of MVA and/or MVA-vectored recombinant HIV-1 vaccines. We describe the frequency of reported cardiac symptoms, cardiac enzyme abnormalities, and clinically significant changes in ECGs, in participants who received MVA vaccines compared to placebo recipients. We determine the number of participants who met the CDC-case definition for vaccinia-related myo/pericarditis or who experienced cardiac adverse events related to these study agents, and propose an approach to cardiac surveillance for future clinical trials of highly attenuated vaccinia vectors.

## Methods

**Figure 1:**
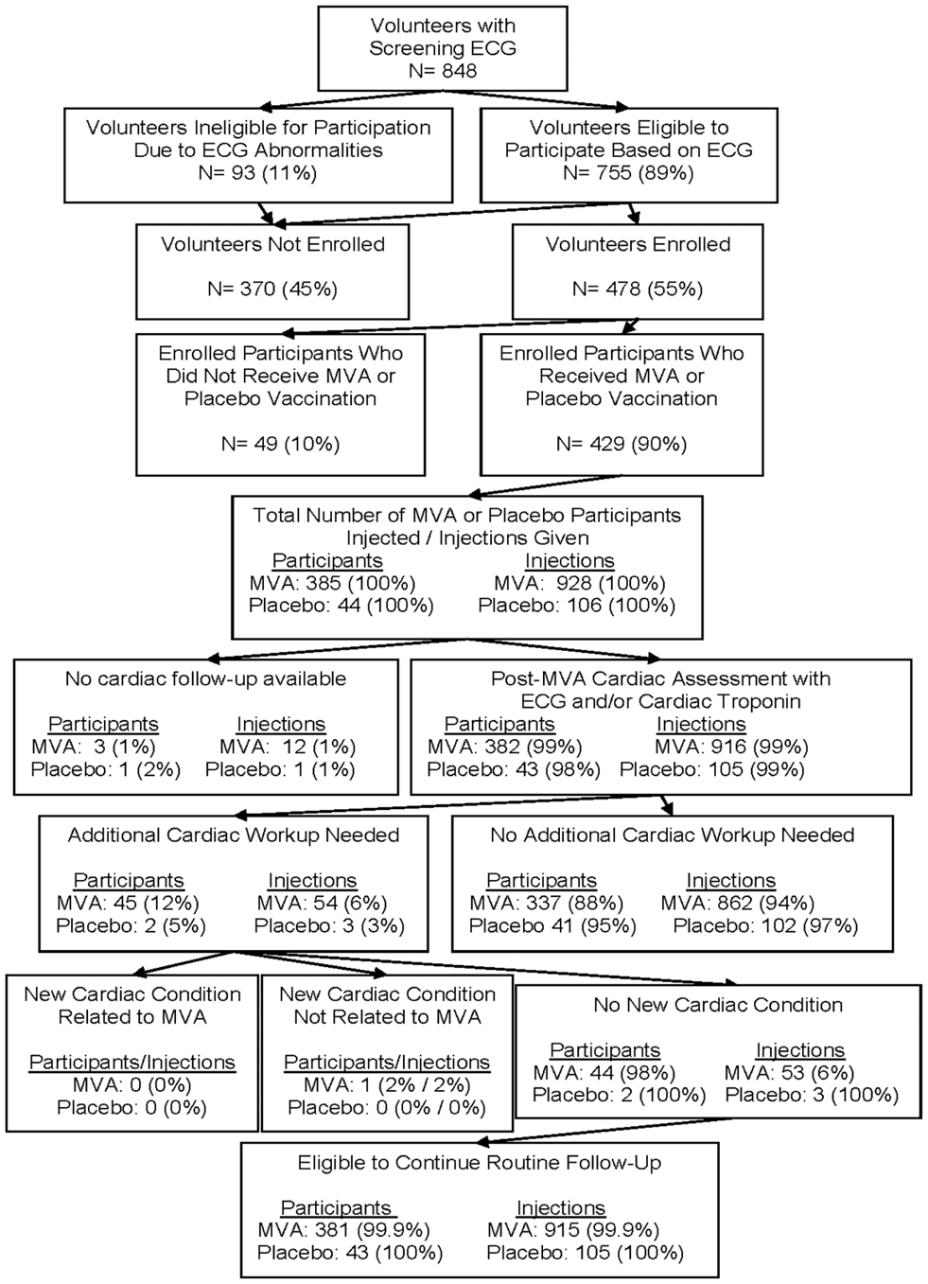
Overview of clinical trial designs. Schema for each of the 6 clinical trials with prospective cardiac safety assessments included in this report, A) US Military HIV Research Program study [NCT00376090] [30], B) National Institutes of Health Division of Microbiology and Infectious Disease/St. Louis University study [NCT00082446] [11], C) Aaron Diamond AIDS Research Center/International AIDS Vaccine Initiative study [NCT00252148] [31], D) HIV Vaccine Trials Network 055 study [NCT00083603] [27], E) HIV Vaccine Trials Network 065 study [NCT00301184] [28], and F) HIV Vaccine Trials Network 067 study [NCT00428337] [29]. ‘Arrows’ indicate time of vaccination; ‘X’ indicates time of routine ECGs; ‘N’ indicates total number of study participants included in this analysis; ‘A:P’ indicates ratio of participants who received active vaccination (A) to placebo (P).

Data from 1 Phase I trial of MVA vaccine (National Institute of Allergy and Infectious Diseases [NIAID] –supported Division of Microbiology and Infectious Diseases Saint Louis University Vaccine and Treatment Evaluation Unit [SLU-DMID] [11]) and 5 Phase I trials of MVA-HIV vaccines (NIAID-supported HIV Vaccine Trials Network [HVTN] [27]–[29], US Military HIV Research Program [MHRP] [30], and the Aaron Diamond AIDS Research Center, in collaboration with the International AIDS Vaccine Initiative [ADARC-IAVI] [31]) were analyzed (Figure 1). All clinical trials were approved by the sites’ institutional review boards and institutional biosafety committees.

### Eligibility Criteria and Follow-up

All six protocols enrolled healthy HIV-negative participants with similar eligibility criteria and post-vaccination cardiac surveillance. Participants were required to have a normal baseline ECG and cardiac troponin I. Exclusionary ECG findings encompassed: 1) conduction disturbance (complete left or right bundle branch block, intraventricular conduction disturbance with QRS >120 ms, AV block of any degree, and QTc prolongation >440 ms; 2) repolarization (ST segment or T wave) abnormality; 3) significant atrial or ventricular arrhythmia, including frequent ectopy (e.g., 2 premature ventricular contractions in a row); and 4) evidence of past myocardial infarction. All ECGs were obtained with GE MAC 1200 ECG machines (GE Healthcare, Chalfont St. Giles, UK), and transmitted electronically to the Saint Louis University Core ECG Laboratory for interpretation. People with any history of, or known active cardiovascular disease, stroke/transient ischemic attack, or risk factors for cardiac disease (2 or more of hyperlipidemia, hypertension, tobacco use, family history of cardiac disease) were excluded.

Participants were vaccinated with MVA or MVA-HIV-1 recombinants or placebo according to protocol-specific vaccination schedules and were questioned about interval symptoms suggestive of myo/pericarditis (such as chest pain, shortness of breath, palpitations, unexplained fatigue, fever or flu-like symptoms) at all visits. Additional routine assessment of serum troponin I was conducted for all participants 2 weeks after each MVA vaccination and all had routine ECGs 2 weeks after initial MVA vaccinations (plus other per protocol time-points indicated in Figure 1). The two-week post-vaccination time-point was chosen for routine cardiac safety assessments because it corresponded well with the CDC case-definition of myo/pericarditis following smallpox vaccination (4–30 days post-vaccination) [32]. MVA dosage ranged from 1×10^6^ to 1×10^9^ pfu/ml and was administered intramuscularly in all studies, but also subcutaneously in the SLU-DMID protocol and intradermally in the MHRP protocol. Symptoms suggestive of myo/pericarditis reported at or between visits were promptly evaluated (symptom-driven cardiac investigations) by ECG and troponin I (some studies included creatine phosphokinase-MB [CK-MB]). If suspicion of cardiac involvement remained after initial work-up, participants were referred to a cardiologist for further evaluation.

### Data Management, Analysis Plan and Statistics

Cardiac safety data from the four research programs were merged into a central database which included, 1) participant demographics, 2) results of routine ECGs and cardiac troponin I testing performed 2-weeks post-MVA injections, 3) cardiac symptoms reported at 2-week post-vaccination visits and 4) results of additional symptom-driven cardiac investigations, conducted at any other time during the studies.

Vaccinations for which 2-week follow-up data was not collected due to missed visits or visits outside the 30-day post-vaccination window were excluded from this analysis. If the ECG was repeated due to artifact, data were included only if the repeat test was within 30 days of vaccination. Data obtained after vaccinations with other live-vector vaccines were excluded. As cardiac and ECG abnormalities were uncommon in these populations, data was pooled across studies to gain efficiency in measuring outcomes. Demographics, ECG and non-ECG cardiac assessments were summarized using proportions. Unadjusted Fisher’s exact tests were used to compare treatment arms for each of the cardiac indicators. All analyses were performed in SAS.

## Results

### Participant Demographics

The demographic data of participants with cardiac assessments are shown in Table 1. Age and gender were balanced between treatment arms of a given study. The mean age ranged from 24–33 years, with MHRP having slightly older participants and the DMID and MHRP studies had slightly more males than females (62–67% male). The majority of trial participants were Caucasian, except for the MHRP trial, in which the majority were African-American. Only one trial included participants who previously received smallpox vaccination; eight (17%) and two (18%) such participants in the ADARC-IAVI trial received MVA and placebo, respectively.

**Table 1:** Demographics of participants with cardiac safety assessments.

		MHRP		ADARC-IAVI			SLU-DMID		HVTN			Total		
		Vaccine	Placebo	Vaccine	Placebo		Vaccine	Placebo	Vaccine	Placebo		Vaccine		Placebo
**N**		33 (9%)	6 (14%)	46 (12%)	11 (26%)		74 (19%)	–	229 (60%)	26 (60%)		382 (90%)		43 (10%)
**Age in years**	Mean (SD)	31 (8.1)	33 (6.8)	28 (7.6)	26 (6.4)		24 (3.7)	–	25 (4.9)	27 (5.8)		26 (5.7)		27 (6.3)
	Range	(18, 48)	(22, 41)	(19, 52)	(18, 40)		(18, 33)	–	(18, 40)	(19, 39)		(18, 52)		(18, 41)
**Gender**	Male	22 (67%)	4 (67%)	23 (50%)	6 (55%)	46 (62%)		–	109 (48%)	12 (46%)	200 (52%)		22 (51%)	
	Female	11 (33%)	2 (33%)	23 (50%)	5 (45%)	28 (38%)		–	120 (52%)	14 (54%)	182 (48%)		21 (49%)	
**Race**	Caucasian	11 (33%)	0 (0%)	25 (54%)	6 (55%)	67 (91%)		–	178 (78%)	21 (81%)	281 (74%)		27 (63%)	
	African American	18 (55%)	6 (100%)	10 (22%)	1 (9%)	4 (5%)		–	30 (13%)	3 (12%)	62 (16%)		10 (23%)	
	Asian	3 (9%)	0 (0%)	3 (7%)	1 (9%)	1 (1%)		–	3 (1%)	0 (0%)	10 (3%)		1 (2%)	
	Native American/Alaskan Native	0 (0%)	0 (0%)	1 (2%)	0 (0%)	0 (0%)		–	2 (1%)	0 (0%)	3 (1%)		0 (0%)	
	Hawaiian/Pacific Islander	0 (0%)	0 (0%)	1 (2%)	0 (0%)	1 (1%)		–	8 (3%)	1 (4%)	10 (3%)		1 (2%)	
	More Than One Race	1 (3%)	0 (0%)	0 (0%)	0 (0%)	1 (1%)		–	8 (3%)	1 (4%)	10 (3%)		1 (2%)	
	Unknown or Not Reported	0 (0%)	0 (0%)	6 (13%)	3 (27%)	0 (0%)		–	0 (0%)	0 (0%)	6 (2%)		3 (7%)	
**Prior smallpox vaccination**	Yes	0 (0%)	0 (0%)	8 (17%)	2 (18%)	0 (0%)		–	0 (0%)	0 (0%)	8 (2%)		2 (5%)	
	No	33 (100%)	6 (100%)	38 (83%)	9 (82%)	74 (100%)		–	229 (100%)	26 (100%)	374 (98%)		41 (95%)	

### Participant Flow

**Figure 2:**
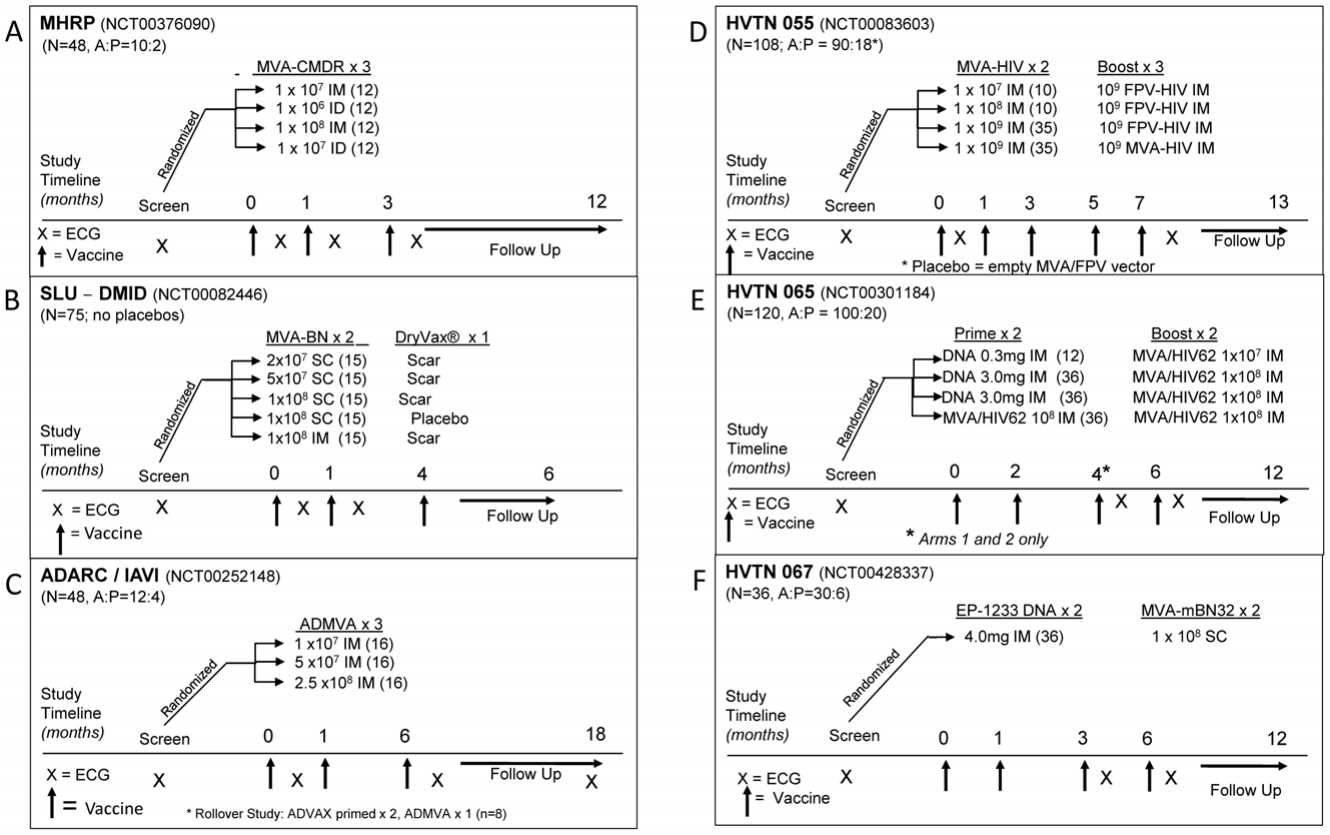
Participant Flow. Flow diagram of participants screened, enrolled and followed with prospective cardiac safety assessments among the 6 trials included in this report. ‘MVA’ indicates MVA alone or an MVA-HIV recombinant candidate vaccine, whereas ‘Placebo’ was a buffered sterile saline solution.

A total of 848 individuals across all trials had screening ECGs for eligibility (Figure 2); 11% were excluded due to baseline ECG findings. Of those meeting ECG eligibility, 478 (55%) enrolled in a trial; of these 429 (90%) participants received MVA, an MVA-HIV recombinant vaccine, or saline at 1 to 5 time-points (median = 2). Ninety-nine percent of these participants had cardiac follow-up. In total, 916 MVA-containing and 105 placebo vaccinations were administered to 382 and 43 participants, respectively.

Symptom-driven cardiac investigations apart from routine 2-week post-vaccination time-points were initiated by at least one of three conditions: 1) volunteer report of clinical symptoms suggestive of myo/pericarditis, 2) an increase in laboratory markers, and/or 3) a significant change on routine ECG. Overall, 47 participants had symptom-driven cardiac investigations, including 12% (45/382) of MVA-vaccinated participants, versus 5% (2/43) of placebo recipients (p = 0.20). Among MVA recipients, 22 investigations were for reported cardiac symptoms, 19 for ECG/laboratory changes, and 4 for cardiac symptoms with an ECG or laboratory change. The two placebo recipients had investigations for cardiac symptoms. In most cases, the repeat ECG and troponin were normal, however 18 participants were referred for more intensive cardiac evaluation: 10 who had symptoms alone, 1 for abnormal troponin, and 7 for ECG changes, which resulted in 15 echocardiograms, 3 Holter monitor tests, 2 cardiac MRIs, and one thallium treadmill stress test being performed. Subsequently, only one participant in the MVA group was diagnosed with a cardiac condition, supraventricular tachycardia (SVT), which was an undisclosed pre-existing condition (see below). Ultimately, no participant was diagnosed with myo/pericarditis.

### Solicited Symptoms Suggestive of Myocarditis or Pericarditis

The percentage of participants with cardiac symptoms suggestive of possible myo/pericarditis 2 weeks post-vaccination was 17.8% (68/382) among MVA recipients and 7.0% (3/43) among placebo recipients (p = 0.084) (Table 2). The most common symptoms in MVA versus placebo recipients were fatigue, chest pain and flu-like symptoms, but only fatigue was statistically significant (p = 0.044). There was no clear relationship between any individual symptom and timing of vaccination (Table 2). Of the 12 participants with chest pain, none had cardiac troponin elevations and 2 had significant ECG changes from baseline (one with incomplete right bundle branch block and one with voltage criteria for left ventricular hypertrophy). In addition, 3 participants reported chest pain at days 7, 24 and 36 days following vaccination, respectively, all without ECG or troponin changes. In all, 7 participants with chest pain, including the 2 with ECG changes, were further evaluated by echocardiography without significant findings.

**Table 2:** Cross-sectional frequency of solicited, self-reported symptoms suggestive of myo/pericarditis at visits 2 weeks post-vaccination/boost.

Symptom	Random-ization^a^	Number of Volunteers With Symptom	Percentage with Symptom	Days post Vaccination Symptom First Reported^b^	Duration of Symptom (days)^b^	p-value (MVAvs. Placebo)
**Any Cardiac Symptom**	MVA	68	17.8%	2.9±4.8	6.8±12.0	–
	Placebo	3	7.0%	7.3±7.8	4.3±2.5	0.084
**Fever**	MVA	6	1.5%	4.7±5.1	1.6±0.9	–
	Placebo	1	2.3%	2±0	1±0	0.53
**Chest Pain**	MVA	12	3.1%	8.9±6.1	12.0±25.3	–
	Placebo	0	0.0%	n/a	n/a	0.62
**Shortness of Breath**	MVA	7	1.8%	5.8±6.3	3.6±3.0	–
	Placebo	0	0.0%	n/a	n/a	1.00
**Palpitations** c	MVA	2	0.6%	6.5±0.7	42.0±59.4	–
	Placebo	0	0.0%	n/a	n/a	1.00
**Fatigue**	MVA	48	12.5%	1.4±3.3	5.1±6.0	–
	Placebo	1	2.3%	1±0	2±0	0.044
**Flu-like Symptoms** c	MVA	9	2.9%	4.3±4.7	6.2±4.1	–
	Placebo	2	4.6%	10.5±7.8	5.5±2.1	0.63

a total number of participants who received: MVA = 382; placebo = 43.

b expressed in mean ± standard deviation; for participants who had multiple reports of a given symptom, the minimum number of days post-vaccination and maximum duration of symptoms were used.

c palpitations and flu-like symptoms not collected for SLU-DMID study.

n/a = not applicable.

Two MVA recipients reported palpitations at the 2 weeks post-vaccination visits (Table 2), while 4 additional MVA recipients reported palpitations at days 22, 47, 56 (normal echo), and 91 following vaccination and 2 placebo recipients reported palpitations, at day 4 and at days 7 and 67 after injection. All participants had normal troponin values and ECGs were unchanged from baseline, except for one who had an elevated troponin (0.15 mcg/L; normal ≤0.04 mcg/L) with prolonged exercise-induced palpitations 22 days after receiving an MVA-HIV vaccine. This participant underwent cardioversion for SVT and subsequently had a normal ECG, echocardiogram and gadolinium-enhanced cardiac MRI. After recurrent episodes of exercise-induced SVT, the participant underwent radiofrequency ablation of an accessory conduction pathway. Although this event met the case definition of suspected vaccinia-induced myocarditis, the person had failed to disclose similar exercise-induced palpitations prior to enrollment and it was concluded to be a pre-existing condition, unrelated to MVA vaccine.

### Laboratory Analyses

Only 3 (0.8%) MVA recipients, all from one study site, had self-limited mild elevations (0.06–0.08 ng/mL; normal <0.05 ng/mL) of cardiac troponin I values at any time-point, versus none among placebo recipients. One participant was training for a marathon, one had sinus arrhythmia and early repolarization and a third reported dehydration and dizziness due to exertion and alcohol intake (a cardiac MRI with gadolinium 33 days later was normal.) CK values were also checked in some studies and were abnormal in 4% (10/234) and 0% (0/26) of MVA versus placebo recipients, respectively. Their occurrence was distributed across treatments and no abnormal CK-MB values were seen.

### ECG Analyses

New onset abnormalities found at any time on post-enrollment ECGs are shown in Table 3.

**Table 3:** New onset ECG changes from routine and symptom-driven cardiac investigations.

		Vaccine Recipient	PlaceboRecipient	Total	Exact p-value^a^ (MVA vs placebo)
**Total N participants**	No. of participants with analyzable ECG	382	43	425	–
	No. of participants with abnormalities listedin this table	62 (16%)	5 (12%)	67 (16%)	0.51
**ECG events**	Early Repolarization	15 (4%)	1 (2%)	16 (4%)	1.0
	Non-specific ST-T wave changes	11 (3%)	0	11 (3%)	0.61
	Non-specific T wave changes	13 (3%)	1 (2%)	14 (3%)	1.0
	QTc >440 ms	6 (2%)	1 (2%)	7 (2%)	0.53
	Voltage criteria for LVH^b^	7 (2%)	0	7 (2%)	1.0
	Premature atrial contractions	0	1 (2%)	1 (<1%)	0.10
	Other change	15 (4%)	2 (5%)	17 (4%)	0.69

a p-values are calculated from Fisher’s exact test for association between variable and onset.

b Left ventricular hypertrophy.

### QTc

Among the 425 participants, 8 had QTc intervals exceeding the predefined 440 ms upper limit of normal. One participant was inadvertently enrolled with a baseline QTc value of 474 ms; all subsequent QTc intervals in this person were <474 ms. The remaining 7 participants (including one placebo recipient) had QTc intervals exceeding 440 ms only after randomization, but the incremental increases were <60 ms. One participant was taking clonazepam and escitalopram, which are associated with QTc prolongation.

### ST-T Wave Abnormalities

Eleven MVA recipients had new ST-T wave abnormalities after enrollment. Of these, four reported fatigue in the 2-week period following vaccination. Participants were otherwise asymptomatic and troponin results were normal. In all cases but one the ST-T wave changes were described as minor according to Minnesota code criteria. Three participants were evaluated with echocardiography with no significant findings. In one 30 year old hypertensive participant, new asymptomatic ST-T wave changes were observed 2 weeks after a second MVA vaccination and again 4 weeks after the third vaccination. Troponins were normal and the ECG changes resolved within 1 day each time. Six months after the final vaccination the participant reported chest pain, and ST-T wave changes were again seen, with a normal troponin and CK-MB. An exercise SPECT study revealed an abnormal hypertensive blood pressure response to exercise, with nondiagnostic exercise-induced ST segment changes, normal myocardial perfusion and left ventricular function. The cardiologist at the site who evaluated the participant noted that the participant previously had left ventricular hypertrophy documented on an echocardiogram and concluded that the ECG changes and atypical chest pain were due to poorly controlled hypertension. In long term follow-up contacts 1 and 2 years later, the participant has reported no cardiac problems.

### T-wave Abnormalities

Thirteen asymptomatic participants (including 1 placebo recipient) with normal troponin values developed T-wave changes; one also had a new ST segment abnormality (described above). The remaining 12 participants had minor T-wave changes; in one it was present at baseline. Three participants with T wave changes were evaluated with echocardiography with no significant cardiac findings.

## Discussion

This report describes 6 phase I clinical trials administering MVA or MVA-recombinant candidate HIV vaccines to 382 primarily healthy young adult (median age 25 years) participants without a history of cardiac conditions or significant cardiac risk factors. These participants were similar to those in recent US military reports of symptomatic myo/pericarditis with respect to age, rare history of pre-existing heart disease, and the majority having not previously received vaccinia. Unlike the surveillance of the military and civilian smallpox vaccination campaigns, our studies employed prospective cardiac safety monitoring: longitudinal questioning about cardiac symptoms, and monitoring of serial serum troponin levels and ECGs, the latter of which were interpreted at a single ECG facility. These prospective assessments allow optimal evaluation of the potential of MVA vaccination to induce both subclinical and clinically apparent myo/pericardial involvement.

Among the 6 studies, 916 MVA vaccinations were administered, of which 382 were primary vaccinations. No cases of confirmed symptomatic or subclinical myo/pericarditis were detected. All participants were selected to be at low-risk for cardiac disease and were questioned regularly about cardiac symptoms of any degree or duration during the studies. In spite of their good general health, 17.8% of vaccinees, and 7.0% of placebo recipients (16.7% of participants overall) reported at least one occurrence of symptoms possibly indicative of mild myo/pericarditis at routine visits. Although self-limited fatigue was more frequently reported by MVA recipients than placebo recipients, most were from a single study that did not have a placebo group and were not indicative of cardiac involvement [11], [33] (adjustment for study effect was not done due to the lack of events reported in two studies). Of note, no one in any of the 6 studies reported symptoms highly suggestive of myo/pericarditis, such as dyspnea interfering with activity or positional pleuritic chest pain.

Troponin I levels were within normal limits in all participants except 3 who had transient mild elevations. In one the elevation was concluded to be due to distance running, which has been reported to cause minor asymptomatic troponin I elevations [34]; in another it normalized within 2 days before any additional investigation; and in the third myo/pericarditis was ruled out by a normal cardiac MRI scan. Importantly, even in this healthy young volunteer population minor non-specific abnormalities were frequently seen on ECGs, which on occasion led to additional cardiac diagnostic testing. Moreover, 93 of 848 (11%) of potential participants screened for the studies were excluded due to ECG abnormalities that could complicate the assessment of myo/pericarditis by ECG criteria.

As mentioned, the reported incidence of symptomatic myo/pericarditis after receipt of replication-competent vaccinia in the US military and civilian vaccination campaigns varied between 1 case per 8065 and 182 vaccinees, respectively. However, it is possible that additional cases of mild myo/pericardial involvement occurred that escaped detection. The best estimate of the incidence of subclinical myo/pericarditis can be derived from clinical trials conducted by Acambis, Inc. (now Sanofi-Pasteur), in which single vaccinations with ACAM2000 vs DryVax were administered with prospective cardiac safety monitoring [23]. These studies identified 10 cases of myo/pericarditis among 1675 primary vaccinees (1 case per 168 vaccinees). Seven of the 10 participants had received ACAM2000 (incidence of 5.73 events/1000 vaccinations,) whereas 3 had received DryVax (10.38 events/1000 vaccinations). Of note, 4 of 7 ACAM2000 recipients and 2 of 3 DryVax recipients with myo/pericarditis were asymptomatic, with abnormalities found only on ECGs and/or cardiac enzyme testing. Thus, this study indicated that for every case of symptomatic myo/pericarditis after receiving replication-competent vaccinia, there could be one or more additional subclinical cases.

Although our report provides information from the largest collection of MVA studies monitored prospectively with similar methods, our experience is still too limited to completely rule out rare events in healthy people that may be observed with more extensive experience or in people with pre-existing cardiac disease. Our finding of no events in 382 MVA recipients has a 2-sided upper 95% confidence bound of 0.96% (9.6 cases/1000 vaccinees or 1 case per 104 vaccinees), which is not significantly different than the rate reported by Acambis of 1 case per 168 vaccinees (p = 0.22). However, a number of studies with attenuated recombinant poxvirus vaccines (MVA or NYVAC), conducted without prospective monitoring of ECGs or cardiac enzymes, have been reported over the past decade and none have reported events consistent with myo/pericarditis [12]–[16], [35]–[40].

The use of attenuated forms of poxviruses including MVA and NYVAC is likely to increase over the coming decades, either for protection from variola as a bioweapon, or as recombinant vaccines for infectious agents such as HIV, malaria and tuberculosis. Although limited by small numbers of MVA recipients, our report demonstrates that MVA is not associated with asymptomatic or symptomatic myo/pericarditis at an unexpectedly high rate (i.e., more than 1 case per 104 vaccinees per the upper 95% CI bound) compared with replication-competent vaccinia and that solicitation of symptoms that could be caused by myo/pericarditis and conducting serial ECGs in a population who is at low-risk for cardiac disease can lead to a variety of non-specific findings that are not clinically significant. Of note, the ongoing experience of the US DoD program, which has now administered replication-competent vaccinia to over 1.2 million people, provides additional assurance that cardiac involvement is rare and when it does occur it is mild and self-limited in vast majority of cases [18]. Specifically, vaccinia-related sudden cardiac death (SCD), which is the primary safety concern should asymptomatic cases of myo/pericarditis fail to be detected, has not been documented in the DoD experience, although one case of SCD was reported it was confirmed to be due to parvovirus B19 by its detection in cardiac tissue on autopsy [18]. In conclusion, we feel that continued awareness of possible cardiac adverse events related to MVA is prudent; however, sound clinical judgment should be the mainstay of assessment of potential cardiac adverse events among recipients of attenuated poxviruses.

## Supporting Information

Checklist S1 **PRISMA 2009 Checklist.**
(PDF)
